# The Impact of Liver Failure on the Immune System

**DOI:** 10.3390/ijms25179522

**Published:** 2024-09-01

**Authors:** Alicja Dąbrowska, Bartosz Wilczyński, Jakub Mastalerz, Julia Kucharczyk, Julita Kulbacka, Anna Szewczyk, Nina Rembiałkowska

**Affiliations:** 1 Faculty of Medicine, Wroclaw Medical University, Pasteura 1, 50-367 Wroclaw, Poland; alicja.dabrowska@student.umw.edu.pl (A.D.); bartosz.wilczynski@student.umw.edu.pl (B.W.); jakub.mastalerz@student.umw.edu.pl (J.M.); 2 Faculty of Pharmacy, Wroclaw Medical University, Borowska 211A, 50-556 Wroclaw, Poland; julia.kucharczyk@student.umw.edu.pl; 3 Department of Molecular and Cellular Biology, Faculty of Pharmacy, Wroclaw Medical University, Borowska 211A, 50-556 Wroclaw, Poland; julita.kulbacka@umw.edu.pl

**Keywords:** liver cirrhosis, chronic liver failure, immune response, cytokines, chemokines, immunosuppression, innate and adaptive immune response

## Abstract

Liver failure profoundly affects the immune system, leading to dysregulation of innate and adaptive immune response. This review explores the intricate relationship between liver function and immune homeostasis. The role of the liver as a central hub in immune response initiation is elucidated, emphasizing its involvement in hepatic inflammation induction and subsequent systemic inflammation. Cytokines, chemokines, growth factors, and lipid mediators orchestrate these immune processes, serving as both prognostic biomarkers and potential therapeutic targets in liver failure-associated immune dysregulation, which might result from acute-on-chronic liver failure (ACLF) and cirrhosis. Furthermore, the review delves into the mechanisms underlying immunosuppression in liver failure, encompassing alterations in innate immune cell functions such as neutrophils, macrophages, and natural killer cells (NK cells), as well as perturbations in adaptive immune responses mediated by B and T cells. Conclusion: Understanding the immunological consequences of liver failure is crucial for developing targeted therapeutic interventions and improving patient outcomes in liver disease management.

## 1. Introduction

Liver disease remains a prominent issue in the contemporary world. Liver cirrhosis is the final manifestation of hepatic fibrosis, and cirrhosis-associated immune dysfunction (CAID) refers to the unique set of immune changes. It is characterized by systemic inflammation and immune deficiency. Cirrhosis is associated with gastrointestinal system dysregulation, leading to impaired gut function and dysbiosis [1].

Nevertheless, the consequences of cirrhosis are local and systemic, affecting most organs and the immune system. The immune response is attenuated, inevitably resulting in immune deficiency. However, as the ability to combat pathological threats declines, the amount of pro-inflammatory cytokines causing systemic inflammation rises [2]. Furthermore, the severity of those processes is not only usually progressive but also associated with the cirrhosis stage [3]

Acute chronic liver failure (ACLF) is a term used to describe a unique syndrome observed in patients with acutely decompensated chronic liver disease. Its occurrence relates to multi-organ dysfunction and unfavorable short-term survival [3]. There is no single recognized definition of ACLF. Significant associations and societies involved in the research of liver diseases have coined their criteria [4]. It leads to missed diagnoses and problems with incorporating proper treatment. However, most researchers agree that it is a severe form of decompensated liver cirrhosis and is associated with a systemic inflammatory response. ACLF is frequently precipitated by some intrahepatic conditions (alcoholic hepatitis, HBV reactivation), extrahepatic conditions (bacterial infection, gastrointestinal bleeding), or both [5]. The predominant precipitating factor exhibits local and definitional variability, although alcohol consumption emerges as the foremost cause.

The liver is essential for immune regulation, producing proteins and cells that manage responses to antigens. Liver failure disrupts this balance, leading to pathological inflammation and contributing to cirrhosis. This review examines the immune consequences of cirrhosis and ACLF, focusing on recent clinical markers and therapeutic targets [6,7]. 

## 2. Role of the Liver in Immune Response

The liver plays a highly significant role in the proper functioning of the immune system, being responsible for cytokine and acute phase reactant production. The hepatic artery supplies the liver with blood, however, it provides only a minor part of oxygen and nutrients to that organ. Most of the blood entering the liver comes from the portal vein. The blood flowing in it is rich in nutrients from the intestines, however, portal blood also contains pathogens and microbe-derived molecules that have passed through the intestinal epithelium [8]. Thus, the liver must develop immune tolerance in the face of harmless substances while providing immunosurveillance and neutralizing pathogens and the harmful molecules they secrete [9]. Multiple cell lines unique to the liver and a distinct proportion of residual lymphocytes facilitate this immune system surveillance. 

Kupffer cells (KC) are the most abundant macrophage population residing in the liver. They are immobilized in sinusoidal lumens, constantly investigating the flowing blood in search of threatening pathogens [7]. However, the cells are not only involved in bacterial endocytosis, but their role in regulating the number of neutrophils has also been documented [9,10,11]. Kupffer cells regulate the number of neutrophils, which is crucial to maintaining hepatic homeostasis and responding to liver injury. These resident macrophages detect disruptions in the liver’s normal state and communicate with other cells to orchestrate an immune response. Liver sinusoidal endothelial cells (LSEC) contribute alongside KCs and intra-hepatic myeloid cells to the regulation of T lymphocytes. T cells remain suppressed in normal circumstances, preventing an excess inflammatory response. This state is achieved by constantly producing anti-inflammatory cytokines such as Il-10, low MHC II content on antigen-presenting cells, and low levels of costimulatory molecules [10]. Upon sensing damage, Kupffer cells release chemokines that attract circulating leukocytes, including monocytes, which differentiate into monocyte-derived macrophages within the liver. This recruitment and differentiation process contributes to the repair and regeneration of liver tissue. It helps regulate the infiltration and activity of neutrophils, ensuring a balanced immune response during acute and chronic liver diseases [11,12].

**Figure 1:**
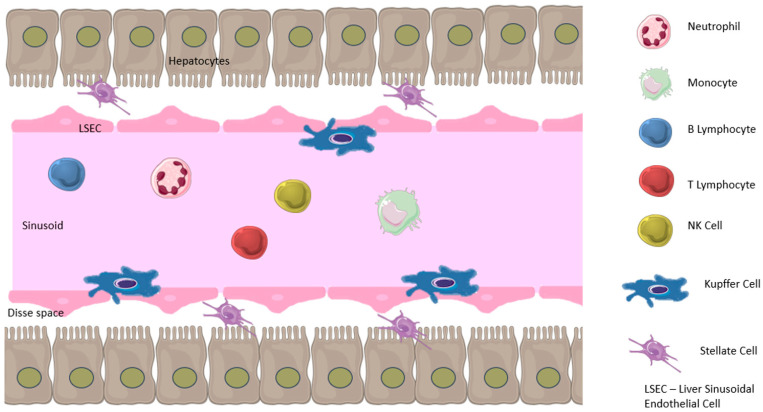
Immune cells in the liver.

The liver is home to most Invariant killer T cells (iNKT) that respond to lipid antigens from both the external and internal environment. They do not require co-stimulation from other cells and can rely solely on TCR interaction with antigens [13]. Immune cells in the liver are shown in the picture (Figure 1).

## 3. Immunometabolism and Mitochondrial Dysfunction in ACLF

Immunometabolism refers to the intricate processes by which immune cells regulate and utilize energy through various metabolic pathways to perform their essential functions, including responding to infections, inflammation, and tissue damage. Under normal conditions, immune cells such as macrophages, T cells, and dendritic cells rely on tightly regulated metabolic processes, including glycolysis, oxidative phosphorylation, and fatty acid metabolism, to meet their energy demands and produce crucial molecules for effective immune responses.

In the context of ACLF, these metabolic processes can become significantly dysregulated. Chronic liver damage, systemic inflammation, and oxidative stress commonly associated with ACLF disrupt normal immunometabolism. This disruption leads to inadequate or excessive immune responses, contributing to chronic inflammation and impaired tissue repair [14]. This dysfunction exacerbates the severity of ACLF, as it impairs the immune system’s ability to manage inflammatory and repair processes effectively.

Mitochondria, the cell’s powerhouses, are central to energy production and cellular metabolism. In ACLF, mitochondrial dysfunction is a prevalent issue. This dysfunction results in impaired oxidative phosphorylation, reduced ATP production, and increased reactive oxygen species (ROS) generation. Studies have shown that, in patients with ACLF, there is a significant decrease in β-oxidation of fatty acids in the mitochondria, leading to reduced oxidative phosphorylation and decreased ATP production [15]. These mitochondrial abnormalities contribute to the systemic energy deficit and oxidative stress observed in ACLF patients, which further aggravates liver injury and promotes multi-organ failure [15,16].

The relationship between mitochondrial dysfunction and immunometabolism in ACLF is bidirectional. On the one hand, impaired mitochondrial function disrupts the metabolic pathways of immune cells, leading to altered cytokine production and immune cell activation. On the other hand, the inflammatory milieu characteristic of ACLF can cause further damage to mitochondria, creating a vicious cycle that exacerbates disease severity [14]. This interconnectedness highlights the critical need for targeted therapeutic strategies aimed at restoring mitochondrial function and modulating immunometabolism to potentially mitigate ACLF progression and improve patient outcomes [17,18,19].

## 4. The Gut–Liver Axis and Initiation of Hepatic Inflammation

The gut–liver axis represents a critical bidirectional communication pathway between the liver and gastrointestinal tract, primarily facilitated by the portal vein. This pathway allows the transportation of various substances, including digestive products, microbial components, and metabolic by-products, from the intestines to the liver [20]. The liver, in turn, influences gut health through bile secretion and immunological factors, which are transported back to the intestines, thereby modulating gut microbiota composition and intestinal immune responses [21].

In a healthy state, this interaction maintains homeostasis. However, in conditions such as cirrhosis, the permeability of the gut membrane significantly increases, a condition often referred to as leaky gut syndrome. This heightened permeability permits the translocation of harmful substances, including bacterial endotoxins like lipopolysaccharides, from the gut into the liver. This influx exacerbates liver injury by promoting systemic inflammation and advancing liver pathology [22].

The immune system plays a crucial role in recognizing translocated substances as threats. Pathogen-associated molecular patterns (PAMPs) and danger-associated molecular patterns (DAMPs), molecules derived from microorganisms and damaged cells, are detected by pattern recognition receptors (PRRs), such as toll-like receptors (TLRs) and NOD-like receptors (NLRs). In cirrhosis, enhanced bacterial translocation and gut dysbiosis lead to a more significant influx of PAMPs into the intestines and surrounding lymphoid tissues. Microbes crossing the intestinal barrier also enter the systemic circulation, triggering low-grade inflammation. Concurrently, DAMPs are continuously released from damaged hepatocytes, further amplifying systemic inflammation and contributing to the progression of liver disease [7,23,24].

## 5. Systemic Inflammation

**Figure 2:**
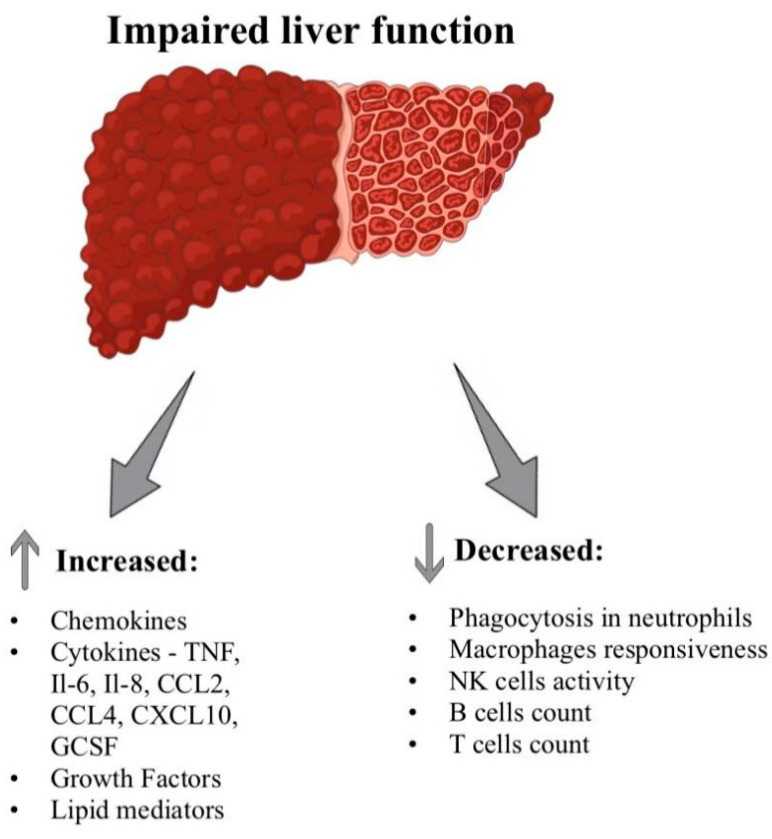
Changes in the release of immune factors in the course of liver disease.

In the course of liver disease, there is both an intense systemic inflammatory response and immunosuppression, which leads to its hyperreactivity and changes in the expression of immune factors (Figure 2). It has been shown that the systemic inflammatory response is the most crucial cause of sudden deterioration in patients who already have chronic liver disease. In the initial stage of ACFL development, pathogens strongly stimulate the immune system and consequent activation, recruitment, and differentiation of effector immune cells. Macrophages play a vital role at this stage. Their population can be divided into M1 macrophages, which participate in antigen presentation and secrete mainly pro-inflammatory cytokines and reactive oxygen species, and M2 macrophages, which secrete mainly anti-inflammatory cytokines [24]. M1 and M2 macrophages will be activated in the early stages of ACLF development. Activation of macrophages (Kupffer cells) present in the liver leads to the recruitment of innate effector cells and liver damage. Circulating monocytes in the blood will also play an important role, as they will infiltrate the liver. As a result of their proliferation, local liver damage can occur, which will induce the secretion of pro-inflammatory cytokines. The expression of progressive inflammation is the increasing secretion of inflammatory mediators, which we can divide into protein mediators (such as cytokines, chemokines, or growth factors) and lipid mediators [25]. Numerous studies have shown that the secretion of inflammatory mediators is directly related to patients’ clinical status and mortality [26]. Moreover, some of these mediators may become therapeutic targets in the future.

## 6. Mediators of Inflammation 

### 6.1. Cytokines

Cytokines are proteins that are critical mediators of inflammation. They are responsible for regulating and determining the nature of the immune response. Depending on which combinations of cytokines are produced, the immune response may develop as cytotoxic, humoral, cellular, or allergic. Once released from immune cells, cytokines bind to specific receptors, initiating a signaling cascade that drives the immune response [27,28]. Cytokines can be broadly divided into pro-inflammatory cytokines, such as TNF-α, IL-1β, and IL-6, and anti-inflammatory cytokines, including IL-10 and IL-4. Both types are involved in the development of liver conditions, such as decompensated cirrhosis and acute-on-chronic liver failure (ACLF) [25]. For example, IL-1β and TNF-α, produced in response to infection and tissue damage, stimulate TLR4, leading to IL-6 secretion—a pleiotropic cytokine that plays a crucial role in acute-phase protein secretion [29]. A study using a mouse model combined with an ethanol-feeding regimen showed that the acute inflammatory response associated with IL-6 can drive the progression from stable chronic inflammation to progressive liver damage [30].

Moreover, the TNF-α signaling pathway may contribute to the induction of hepatocyte apoptosis in ACLF. Despite the elevation of IL-10 levels, a marked imbalance in favor of pro-inflammatory cytokines persists, exacerbating liver damage [31]. Baseline levels of inflammatory markers measured in the plasma of patients on admission are slightly elevated in compensated cirrhosis, significantly increased in decompensated cirrhosis, and peak in ACLF patients, who exhibit “full-blown” systemic inflammation. Compared to controls, patients with decompensated cirrhosis show elevated inflammatory and anti-inflammatory cytokine levels, including IL-6, IL-7, IL-8, IL-10, IL-12, and TNF-α. However, patients who develop ACLF show an opposite pattern, with decreased levels of IL-7, IL-10, IL-12, TNF-α, MCP-1, and IFN-γ, while levels of IL-6 and IL-8 remain elevated [32]. Another group observed a significant increase in cytokines and chemokines in patients with acute decompensated cirrhosis, with a further increase in cytokines in patients with ACLF, particularly those involved in the innate immune response. The overwhelming secretion of inflammatory mediators in ACLF is so pronounced that it is often referred to as a “cytokine storm”, a phenomenon also observed in sepsis and severe COVID-19 infections [33,34].

In addition to these well-known cytokines, it is worth highlighting the potential of IL-22 and its fusion protein, IL-22FC, in modulating the immune response and promoting tissue repair. IL-22, a member of the IL-10 cytokine family, is crucial to mediating communication between immune cells and epithelial tissues. It primarily signals through the IL-22 receptor, expressed on non-hematopoietic cells, such as epithelial and liver cells, rather than immune cells. When IL-22 binds to its receptor, it activates downstream signaling pathways, particularly the STAT3 pathway. This activation leads to the transcription of genes involved in tissue protection, regeneration, and the maintenance of barrier function. These anti-apoptotic, proliferative, and antimicrobial effects make IL-22 an attractive candidate for therapeutic strategies, especially in tissue damage and inflammation conditions. Thus, IL-22 and IL-22FC offer promising therapeutic potential in treating inflammatory diseases and tissue injury conditions [35].

#### 6.1.1. Cytokines as Prognostic Biomarkers

Cytokine levels correlate with clinical status and patient mortality, making cytokines good candidates to be prognostic biomarkers. A study of 412 patients with hepatitis B virus-related acute-on-chronic liver failure (HBV-ACLF) showed a positive correlation between high serum IL-6 levels and prognosis. In a retrospective analysis, the predictive value of IL-6 levels on 90-day mortality in patients with ACLF was similar to that of MELD (Model of End-Stage Liver Disease) or Meld-Na. It was higher than CRP and serum leukocyte levels [36]. Moreover, IL-6 has recently been shown to be an independent risk factor for death after 28, 90, and 180 days in patients with liver failure [37]. Moreover, the predictive value of serum IL-6 concentration in the development of overt hepatic encephalopathy within 180 days was higher than the efficacy of MELD, especially for patients who had not previously developed overt hepatic encephalopathy [38]. In contrast, a study of patients with esophageal variceal bleeding showed an association between the incidence of infection and death in these patients, and levels of high mobility protein group 1 (HMG1) and IL-6. Also, patients with clinically significant portal hypertension (≥10 mmHg) had elevated serum levels of IL-6 and IL-18. More recently, IL-6, IL-22, interferon-α2, soluble TNF receptor 1, lipocalin-2, and α-fetoprotein have been linked to mortality within 28 days in patients with severe alcoholic hepatitis, and IL-6, IL-13, and endotoxin levels to mortality within 90 days. Moreover, a parameter composed of IL-13 levels and age was better at predicting 90-day mortality in patients with AH than MELD [39]. Low levels of stem cell factor (SCF), low levels of essential fibroblast growth factor (bFGF), and high levels of IL-13 have also been shown to correlate with an increased risk of ACLF [40]. IL-6, initially identified as a key factor in antibody production, has since been recognized as a critical pathway in immune regulation, with its dysregulation implicated in numerous diseases. Targeted therapies that inhibit IL-6 signaling have revolutionized treatment for various conditions, including rheumatic diseases like rheumatoid arthritis, and are being explored for expansion into other areas such as uveitis, neuromyelitis optica, and COVID-19 pneumonia [41].

#### 6.1.2. Cytokines as Potential Therapeutic Targets

**Figure 3:**
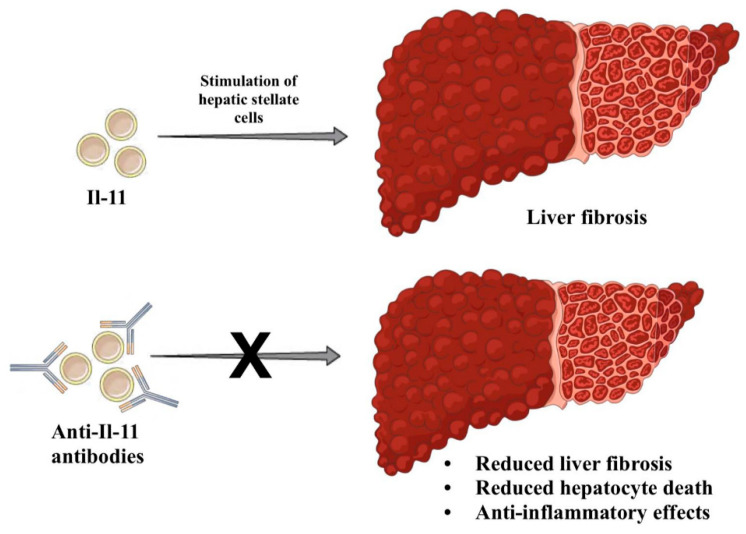
Anti-Il-11 antibodies reduce fibrosis and hepatocyte death, and have anti-inflammatory properties.

Due to the high mortality rate among patients who develop ACFL, and the lack of effective therapy, new therapies, including those based on interference with cytokine signaling, are being sought all the time. Based on a model of severe liver impairment, impaired liver regeneration processes have been shown to result from a shift in activation of the IL-6/STAT3 pro-regenerative pathway to the IFN-γ/STAT1 anti-regenerative pathway, which in turn was related to the inability of Kupffer cells to produce IL-6. Therapy with IL-22Fc reversed this switch in mice and may have therapeutic potential for humans in the future [42,43]. Recently, a phase II study was conducted evaluating the therapeutic potential of F-652, a type of IL-22Fc, in the treatment of patients with alcohol-associated hepatitis. F-652 has been shown to improve patients’ prognosis, as demonstrated by a reduction in Lille and MELD scores, and a decrease in serum cytokine and chemokine levels [44]. Manipulating the signaling of other cytokines, such as IL-6 and IL-11, may also be an important therapeutic option for treating ACLF in the future. However, for now, there are many obstacles in the way. For example, IL-11 has also been shown to have pro-inflammatory and anti-regenerative effects [45,46]. It has been observed that therapy with antibodies that block IL-11 signaling leads to reduced fibrosis, has anti-inflammatory effects, and reduces hepatocyte death in mice with diet-induced steatohepatitis [47] (Figure 3). The use of anti-IL11 therapy is being considered in many diseases, including the treatment of acute and chronic kidney disease [48]. Treatment possibilities for various immunological dysfunctions are shown in Table 1.

### 6.2. Chemokines

Chemokines (chemotactic cytokines) are a family of small and soluble proteins that act as signaling molecules and function by binding to G-protein-coupled receptors (GPCRs) on the cell surface [49]. They play a crucial role in immune system homeostasis and participate in developing immune and inflammatory responses by stimulating leukocyte migration [50]. Due to differences in the number and position of the N-terminal cysteine residues, chemokines can be divided into four subfamilies: the CXC group, the CX3C group (CX3CL1 or fractalkine), the (X)C group, and the CC group [51]. The increased secretion of chemokines such as CXCL10, CCL2, CCL4, and IL-8 have been observed in both cirrhosis decompensation and ACFL [31,32]. IL-8 is secreted by hepatocytes, stellate cells, and Kupffer cells, among others, and its levels are associated with the prognosis of patients with ACLF [52]. The gene encoding IL-8 is activated in response to IL-1 and TNFα due to the synergistic action of NF-κB in combination with AP-1 or C/EBP [53]. Recently, it has also been suggested that high levels of IL-1β in the serum of ACLF patients may promote IL-8 expression in human umbilical cord mesenchymal stem cells (hUC-MSCs) via the NF-κB signaling pathway, thereby reducing the therapeutic effect of hUC-MSCs on ACLF [54]. It has also been reported that IL-8 can induce the conversion of mature hepatocytes toward a cholangiocyte phenotype [55]. Retrospective analysis showed that IL-8 may be an independent predictor of 28-day mortality in ACLF-HBV patients, but not mortality at 90 and 180 days [56]. More recently, CXCL2, IL-8, total bilirubin, and age were isolated as independent risk factors for poor prognosis in HBV-ACLF patients, and an immunological predictive model was developed based on them, which had a higher prognostic value than Chronic Liver Failure Consortium (CLIF-C) ACLF, MELD, and MELD-Na [57]. In another study, HBV-ACLF patients had statistically significantly higher serum CXCL1 levels compared to healthy subjects, patients with chronic HBV infection, and patients with HBV-compensated cirrhosis [58]. Studies using a mouse model of ACLF have shown that CXCL1 is involved in the mobilization of ACLF neutrophils, and its knockdown leads to reduced neutrophil infiltration, the release of fewer reactive oxygen species, and inhibition of hepatocyte apoptosis, resulting in reduced liver damage and inflammation [59]. Further studies are needed to determine whether CXCL1 can become a prognostic marker and therapeutic target [58]. Elevated IL-8, CXCL9, and CXCL10 levels were associated with shorter survival of cirrhotic patients undergoing transjugular intrahepatic portal-systemic shunt (TIPS). Moreover, CXCL10 has also been linked to the incidence and mortality of ACLF [60,61,62]. Miao Huang et al. recently identified C-C motif chemokine ligand 5 (CCL5) as an essential factor leading to reduced liver regenerative capacity. CCL5 induced the transition of macrophages into a pro-inflammatory phenotype via the Forkhead box O (FoxO) 3a pathway, which led to reduced hepatocyte growth factor (HGF) production. CCL5 may be a promising therapeutic target for treating post-hepatectomy liver failure (PHLF) [63].

### 6.3. Growth Factors

In addition to cytokines and chemokines, granulocyte-macrophage colony-stimulating factor (GM-CSF) and granulocyte colony-stimulating factor (G-CSF) were abundant in the serum of ACLF patients [31]. They are crucial for differentiating and activating monocytes and neutrophils [64]. In the liver, they induce progenitor cell proliferation [65]. In the case of ACLF, it was reported that G-CSF administration was associated with improved patient survival, which was most likely related to the restoration of immune function and reduced risk of infection [66]. Based on ACLF models, it has been shown that the use of G-CSF and a TLR4 inhibitor leads to the inhibition of inflammation, promotes liver regeneration, and may reduce mortality in ACLF [67]. The transforming growth factor β1/interleukin-31 (TGF-β1/IL-31) pathway has also been shown to correlate with the degree of liver damage and the worse prognosis of patients with ACLF [68]. High fibroblast growth factor 21 (FGF21) levels were observed in cirrhotic patients admitted to the intensive care unit. Moreover, it was higher in patients with ACLF but did not correlate with the severity of the course of ACLF [69].

### 6.4. Lipid Mediators

Lipid mediators are biologically active lipids that mainly affect numerous cellular processes by binding to G-protein-coupled receptors [70]. In the course of liver diseases, there is an imbalance in the secretion of lipid mediators, including a disruption between pro-inflammatory and anti-inflammatory mediators [71]. Analyzing the plasma of 200 patients with decompensated cirrhosis with or without ACFL for as many as 100 lipid mediators, 16 lipid mediators associated with the patients’ condition were identified. Moreover, levels of Leukotriene E4 (LTE4) and 12-Hydroxyheptadecatrienoic acid (12-HHT) distinguished patients with ACLF from those without ACLF. In addition, LTE4 levels were associated with inflammatory markers, non-apoptotic cell death, and short-term patient mortality [72]. LTE4, like other leukotrienes, is a known mediator of the inflammatory response [73]. Moreover, analysis of albumin-associated lipid mediators showed that patients with acute, uncompensated cirrhosis at risk of disease progression to ACFL have low levels of anti-inflammatory lipid mediators. Prostaglandin (PG) E2 was absent from the albumin of patients with acute uncompensated cirrhosis, suggesting that it is mainly present in this group of patients in a form separated from albumin [74]. The survival of patients with ACLF was also linked to changes in the profile of levels of pro-secretory lipid mediators [75]. Previous studies have also reported that PGE2 is associated with immune deficits in patients with acute decompensated cirrhosis, although the literature data on the subject are divergent [72,76]. PGE2 analysis, long available through ELISA or HPLC, has been central to understanding lipid mediators. However, recent lipidomic studies have expanded this understanding by revealing complex lipid profiles associated with cirrhosis and its progression to ACLF [77]. Specifically, these studies identified distinct lipid signatures—sphingomyelins for acute decompensation and cholesteryl esters for ACLF—highlighting the dynamic nature of lipid changes during disease progression. PGE2 levels were higher in HBV-ACLF patients than in healthy controls and patients with stable viral hepatitis. Moreover, it was shown that increased serum PGE2 levels were associated with a higher risk of infection in patients with ACLF [78]. Moreover, reduced prostaglandin E receptor 2 (EP2) expression on CD8+ T cells was observed in HBV-ACLF patients. Altered PGE2-EP2 was associated with excessive inflammation and stimulation of cells of the acquired immune system in response to LPS and Escherichia coli infection [79]. Albumin infusions have also been shown to promote the reversal of immune dysfunction by binding and inactivating PGE2 [80]. In addition, increased PGE2 levels in patients with acute, uncompensated cirrhosis and who received albumin may be associated with a higher risk of inflammation during the first few days after hospitalization [75]. On the other hand, based on a mouse model, it was also shown that PGE2 secreted by MSCs showed therapeutic potential in treating acute liver failure. PGE2 blocked TGF-β-activated kinase 1 and activated the NLRP3 inflammasome in liver macrophages, reducing cytokine secretion. In addition, PGE2, through the STAT6 and mTOR pathway, induced the transition of macrophages to an anti-inflammatory (M2) form, contributing to reducing hepatocyte death and inflammation [81].

## 7. Immunosuppression

Liver failure leads to systemic inflammation and strongly affects immune responsiveness. Due to immune dysfunction, innate immune paralysis, disturbance in immune tolerance, and decreased innate and adaptive responses occur. Consequently, those patients develop immunosuppression, which might lead to opportunistic infections and lowered response to vaccinations. This chapter discusses changes in immune cell counts and cellular pathway pathologies (Table 2, Table 3 and Table 4).

### 7.1. Innate Immune Response 

#### 7.1.1. Neutrophils

The liver plays a crucial role in the innate immune response, thus, liver failure might significantly impact its responsiveness. It has been well known since the 1970s that cirrhosis impairs neutrophil phagocytosis, chemotaxis, superoxide production, and attenuated degranulation responses [82,83,84,85].

**Figure 4:**
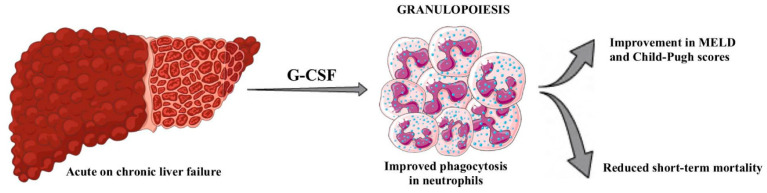
The figure presents the role of the G-CSF in granulopoiesis and its effect on the clinical course of liver failure.

More importantly, correlations have been found between neutrophils in patients with ACLF and increased mortality [86]. There has also been a study comparing neutrophils from patients with cirrhosis to healthy controls, which has shown decreased expression of a neutrophil adhesion receptor CD62L and reduced transendothelial migration [87]. Exposure to G-CSF is proven not only to significantly improve phagocytic and bactericidal function in neutrophils from acute liver failure patients ex vivo, but also might reduce short-term mortality in patients with ACLF [87,88,89]. Furthermore, a meta-analysis from 2023 carried out by Konstantis et al. has shown that administering G-CSF might lead to improvements in overall survival, liver function, and prognosis, as evidenced by the improvement in the MELD score [90] (Figure 4). On the contrary, another study suggests no clear conclusions regarding the usefulness of G-CSF in ACLF. However, survival benefits were observed in Asian patients [91].

**Table 2:** Changes in immune cell count changes and neutrophil-related pathway pathologies.

Neutrophils			
Author (Year)	Article Type	Number of patients	Conclusions
Mookerjee R. P. et al. (2007) [86]	Research Article	63 patients with alcoholic cirrhosis and cirrhosis superimposed on AH	Full activation of neutrophils resulting from the presence of humoral factors such as endotoxin is associated with reversible impairment of their immune response to infection
Fiuza C. et al. (2002) [87]	Research Article	14 patients with liver cirrhosis and 14 healthy controls	Increased neutrophil adhesion to microvascular endothelium and deficient transendothelial migration in patients with liver cirrhosis. G-CSF increases neutrophil transendothelial migration in patients with cirrhosis
Kedarisetty C. K. et al. (2015) [88]	Randomized Controlled Trial	55 patients with decompensated liver cirrhosis	Reduced mortality and reduced symptom severity during 12 months of follow-up in patients treated with G-CSF and darbepoetin α
Konstantis G. et al. (2023) [90]	Systematic Review and Meta-Analysis of Randomized Controlled Trials	421 patients with ACLF	Reduced mortality and improved MELD scores in patients treated with G-CSF. There is no correlation between improved Child–Pugh score or complication rates.
Hou X. et al. (2021) [91]	Systematic Review and Meta-Analysis of Randomized Controlled Trials	479 patients with ACLF	Overall, there is no association between G-CSF treatment and reduced risk of death or complications. Correlation with better survival in the Asian population.

#### 7.1.2. Macrophages 

It is crucial to note that, as cirrhosis or ACLF progresses, immune responses become attenuated and are accompanied by macrophage hyporesponsiveness. There is evidence that the cyclooxygenase (COX)-derived eicosanoid prostaglandin E2 (PGE2) correlates with cirrhosis-associated immunosuppression. Elevated circulating concentrations of PGE2 were found in the plasma of patients with acute decompensation of cirrhosis [76]. Albumin, which reduces PGE2 bioavailability, was decreased in the serum of patients with acute decompensation and appears to have a role in modulating PGE2-mediated immune dysfunction. In vivo administration of human albumin solution to these patients significantly improved the plasma-induced impairment of macrophage pro-inflammatory cytokine production in vitro. Consequently, it creates a therapeutic option—human albumin solution infusions may be used to reduce circulating PGE2 levels and thus reduce the risk of infection in patients with acutely decompensated cirrhosis [76,92,93].

Moreover, several studies have shown that circulating monocytes in patients with ACLF exhibit increased expression of the inhibitory MERTK (Mer receptor tyrosine kinase), which regulates M2 macrophage polarization via the JAK1/STAT6 signaling pathway. This expression is higher than in patients with stable cirrhosis and healthy controls [94]. Furthermore, upregulation of MERTK weakens the antigen presentation function and reduces the secretion of inflammatory cytokines [95]. Recently, a study on mice with ACLF detected changes in MERTK, JAK1/STAT6, inflammatory cytokines, and macrophage polarization markers in vitro and in vivo. Furthermore, treatment with mesenchymal stem cells improved liver function and 48-h survival of ACLF mice, but also alleviated inflammatory injury by promoting M2 macrophage polarization and elevated MERKT expression levels in macrophages [96]. 

#### 7.1.3. Natural Killer Cells (NK Cells)

Constituting as much as half of the resident lymphocyte population in a healthy liver, natural killer cells exhibit a significantly higher abundance than peripheral blood does. Natural killer (NK) cells have cytotoxic and antitumor properties, enabling them to neutralize pathogens and impair the development of neoplasms [97]. Weiss et al. observed in their study a reduced proportion of memory lymphocytes and NK cells in the immune cell profile of patients with ACLF. The selective depletion of these cells emerged as a significant factor contributing to systemic immunosuppression [98]. In patients with cirrhosis, NK cell lytic activity is reduced by elevated levels of TGF-β in advanced liver disease in mouse models [99]. Another critical factor might be suppression of NKG2D, an inhibitory receptor on the NK cell, which is the likely mechanism by which their cytotoxic activity is reduced in chronic liver disease [100].

A different study has shown a possible role of NK cells in HBV-ACLF. Increased counts of CXCL-10 and NK cells were found in the liver, and excessive production of CXCL-10 in the peripheral blood contributed to the apoptosis of NK cells in vitro, as the influence of CXCL-10 and NK cells on each other might mediate the unbalanced distribution of NK cells. Moreover, the decrease in NK cells was associated with the level of HBV DNA and disease severity and had good predictive performance in predicting the outcome of patients with HBV-ACLF through AUROC analysis [101].

**Table 3:** Changes in immune cell count changes and Natural killer cells-related pathway pathologies.

Natural Killer Cells (NK Cells)			
Author (Year)	Article Type	Number of Patients	Conclusions
Weiss E. et al. (2021) [99]	Research Article	67 patients with decompensated cirrhosis (including 35 critically ill patients with ACLF in the intensive care unit) and 12 healthy subjects	Increased numbers of neutrophils and macrophage M0-like monocytes and decreased numbers of several lymphocyte subsets (including memory lymphocytes) in patients with ACLF
Radaeva S. et al. (2006) [101]	Research Article	Not applicable (mouse model)	NK cells killed activated hepatic stellate cells (HSCs), alleviating liver fibrosis. This process depended on retinoic acid early inducible 1/NKG2D and tumor necrosis factor-related apoptosis-inducing ligand.
Jeong W. Il et al. (2011) [100]	Research Article	Not applicable (mouse model)	Transforming growth factor-β (TGF-β), produced by indirectly activated HSCs, reduced NK cell cytotoxicity, including that targeting HSCs. It has been suggested that retinol/SOCS1/TGF-b metabolites may be therapeutic targets and improve treatment efficacy with IFN-c and NK cell therapy.
Li H. J. et al. (2022) [102]	Research Article	37 HBV-ACLF patients and 13 control subjects	Reduced number of NK cells in the blood of ACLF patients and altered phenotypic and functional profile of NK. The observed association of these disorders with excessive CXCL-10 production

**Table 4:** Changes in immune cell count changes and macrophage-related pathway pathologies.

Macrophages			
Author (Year)	Article Type	Number of Patients	Conclusions
Bernsmeier C. et al. (2015) [95]	Research article	41 patients with ACLF, 9 patients with acute decompensation of cirrhosis without ACLF, 17 patients with cirrhosis without decompensation, 23 patients with acute liver failure, 29 healthy individuals	Patients with ACLF have higher numbers of immunoregulatory monocytes and macrophages expressing MERTK, suppressing the innate immune response to microbes. MERTK inhibitors can restore the production of inflammatory cytokines by immune cells from ACLF patients and may be developed to enhance the innate immune response in these individuals
Kou K. et al. (2022) [96]	Review article	Not applicable (number of patients not mentioned)	Monocytes and macrophages show decreased HLA-DR expression and increased MERTK expression.
Li Z.-H. et al. (2023) [97]	Research article	Not applicable (mouse model)	In ACLF mice, MSCs enhanced liver function and 48-h survival while reducing inflammatory injury by promoting M2 macrophage polarization and increasing Mertk expression levels in macrophages.

### 7.2. Adaptive Immune Response

Acute-on-chronic liver failure (ACLF) occurs in patients who have an acute decompensation of cirrhosis, consequently leading to organ failures and an increased risk of in-hospital mortality. ACLF is connected with systemic inflammation, elevated blood leukocyte counts, increased plasma levels of C-reactive protein, and increased concentrations of cytokines and chemokines [102]. The white blood cell count is a component of the ACLF scoring system developed by the Chronic Liver Failure Consortium, providing an accurate prediction of early mortality in ACLF patients [103]. It has been proposed that in ACLF, peripheral leukocytosis is enriched with effector immune cells with a high potential for inducing tissue damage [31]. Immune cells, their modulators, and their functions are presented below in Table 5. 

#### 7.2.1. B Cells 

Cirrhosis affects the innate immune response and causes dysregulation of the adaptive immune response. Studies have proven decreased numbers of B cells, including the CD5+ subset, among patients with alcoholic liver disease [105]. Weiss E. et al. conducted a study using results of clinical complete blood count measurements and microarray (genomewide) analysis of blood RNA expression in HS and three groups of patients with cirrhosis, comprising AC, AD, and ACLF. The key results showed that patients with ACLF had leukocytosis fueled by increased populations of neutrophils (that had a unique phenotype) and macrophage M0-like monocytes, and, as expected, which will be described later in this paper, decreased lymphocyte count related to a depletion in memory lymphocytes (of the B-cell, CD4 T-cell lineages), CD8 T cells, and NK cells [98].

Another study demonstrated the loss of CD27+ memory B cells, impaired function, impaired IgG production, and reduced allostimulatory capabilities. Hiroyoshi D. et al. found out that, among patients with chronic hepatitis C, only those that have progressed to cirrhosis display a loss of CD27+ memory B-cells with associated functional abnormalities, such as impaired activation, impaired TNFβ and IgG production, and impaired allostimulatory capacity. However, overall immunoglobulin levels are elevated in cirrhotics due to increased levels of pathogen-specific immunoglobulins such as antibodies against Saccharomyces cerevisiae (ASCA) and against Galα1–3Galβ1–3GlcNAc (alpha-Gal) [106]. This study has shown that cirrhosis is associated with profound reductions of CD27+IgM+ B-cells, a subset of memory B-cells thought to be generated in response to T-independent antigens [107]. Dysfunctional B-cell activation in cirrhosis resulting from hepatitis C infection associated with the disappearance of CD27-positive B-cell population.

Furthermore, another study has proven that alcoholics admitted for acute alcoholic liver disease (ALD) had decreased CD5+ count. In addition to the loss of CD5+ B cells, there was a reduction in the percentage of B cells which were CD5- CD45RAhi. This subset appears phenotypically similar to the IgM-producing CD5- CD45RAlo subset and thus may be enriched for autoantibody-producing cells [108]. 

Liver cirrhosis might also influence the efficacy of the hepatitis B vaccination, as shown in numerous studies. Hassnine A. et al. carried out a retrospective observational clinical study on 500 individuals (400 chronic HCV patients and 100 healthy controls), who were divided into five groups: A (control group), B (cirrhotic patient not receiving treatment), C (chronic hepatitis patients receiving treatment), D (cirrhotic patients receiving treatment), and E (HCC patients receiving treatment). HCV infection and its complications, such as cirrhosis, are one of the main risk factors for vaccine hypo-responsiveness, as they found a significantly lower response to HBV vaccination in HCV-infected individuals, with an overall response rate of 80% and on multivariate analyses that included age, gender, cirrhosis, alcohol abuse, and Diabetes Mellitus (DM), only patients with liver cirrhosis were less likely to be reactive [109]. Another study from 2020 retrospectively evaluated all patients with chronic HCV infection at Hennepin County Medical Center in Minneapolis, Minnesota, between 2002 and 2018. The researchers noticed a significantly lower response to HBV vaccination in HCV-infected individuals, with an overall response rate of 79% and a response rate of 50% when the cohort of those who received three or more vaccine doses was assessed, compared to the response rate of 90–98% among the healthy population [110].

#### 7.2.2. T Cells

The activity of T cell populations during liver disease is influenced by various factors, including the extent of gut microbe translocation, the activation of antigen-presenting cells (APCs), and the proliferative capacity of T lymphocytes, which tends to decrease with the progression of cirrhosis [111].

A recent study involving ACLF patients revealed a significant increase in mononuclear myeloid-derived suppressor cells. This expansion reduced T cell proliferation and heightened vulnerability to bacterial infections [112].

Furthermore, an increase in T regulatory cells might be connected with poorer short-term survival in ACLF patients with hepatitis B, which might be used as a therapeutic target in the long term. The restoration of a balanced T_Reg_ to Th17 ratio seems crucial for improved outcomes, and as a consequence, it likely signifies a proper return to immune homeostasis [113].

One of the most recent studies indicates lower expression of HLA-DR, CD86, and CD54 on monocyte-derived dendritic cells in ACLF patients compared to chronic hepatitis B patients and healthy controls. This may relate to higher levels of procalcitonin (PCT), lower levels of albumin, and decreased prothrombin activity. These patients’ T cells also showed lower Ki-67 and interferon-gamma (IFN-γ) production [114]. 

## 8. Conclusions

It is well known that the liver plays a crucial role in the proper functioning of the immune system, as it creates immune tolerance and neutralizes pathogens and their metabolites. The primary causes of cirrhosis are chronic hepatitis B, alcoholic liver disease, chronic hepatitis C, and non-alcoholic fatty liver disease (NAFLD). Each condition raises the risk of infection and compromises the immune system [115,116]. The development of liver conditions, including decompensated cirrhosis and ACLF, involves both anti-inflammatory and pro-inflammatory cytokines. Moreover, ACLF patients exhibit a predominance of pro-inflammatory cytokines, including TNFα and IL-6, along with chemokines, such as IL-8, MCP-1, IP-10, and MIP-1β. The extensive secretion of inflammatory mediators in ACLF is often called a “cytokine storm”. Moreover, cytokines might be used as prognostic markers and potential therapeutic targets.

Regarding the studies, Il-6 is an independent risk factor for death after 28, 90, and 180 days in patients with liver failure [37]. A recent phase II study investigated the therapeutic efficacy of F-652, an IL-22Fc variant, in patients with alcohol-associated hepatitis. While exploring the manipulation of signaling pathways involving other cytokines like IL-6 and IL-11 holds promise as potential therapeutic avenues for future ACLF, further research is essential [117]. 

As versatile signaling molecules, chemokines significantly influence immune responses and inflammatory processes by regulating leukocyte migration through interaction with G-protein-coupled receptors. CXCL2, IL-8, total bilirubin, and age can be independent prognostic factors in HBV-ACLF patients. 

Another potential therapeutic method is exposure to the G-CSF, which has been shown to improve overall survival and liver function, as evidenced by the improvement in the MELD score [90]. Macrophages also become impaired in ACLF patients. 

Immunosuppression is a significant clinical problem in patients with liver failure. Increased CXCL-10 levels promote NK cell apoptosis, which contributes to systemic immunosuppression, and the IgG production and costimulatory functions of CD27 memory B cells are impaired in cirrhosis. In addition, the increase in regulatory T cells is probably linked to worse outcomes in HBV patients. On the other hand, reduced T cell proliferation evoked by myeloid-derived suppressor cells causes patients to be more prone to developing life-threatening infections. This paper discussed the most critical aspects of the immune system in liver failure. However, further research is still needed, as liver diseases are a significant problem in society, and studies over possible markers and therapeutic targets might improve the mortality rate and quality of life of those patients. 

## Figures and Tables

**Table 1:** Treatment possibilities address various immunological dysfunctions.

Impaired Function of Immune System	Treatment Possibilities	Treatment Effects
Activation of IFN-Î^3^/STAT1 anti-regenerative pathway	F-652 (IL-22 Fc)	Reduction in Lille and MELD scores, decrease in serum cytokine and chemokine levels.
Increased levels of IL-11	Anti IL-11 antibodies	Reduced fibrosis, anti-inflammatory effect, reduced hepatocyte death
Increased levels of PGE2	Human albumin solution	Improved macrophage function, Reduced infection risk
Impaired neutrophil phagocytosis, chemotaxis, superoxide production, and attenuated degranulation	G-CSF	Improvement in Child–Pugh and MELD scores reduced short-term mortality

**Table 5:** Immune cells, their effector molecules, and actions [104].

Immune Cells	Effector Molecules	Action
Plasma and Memory B cells	IgA, IgG, IgE	Eliminate antigen
Th1	IFNγ, TNF, IL-2, Lymphotoxin	Pro-inflammatory
Th2	IL-4, IL-5, IL-6, IL-10, IL-13	Pro-inflammatory, Allergy
Th9	IL-9, IL-10, IL-21	Pro-inflammatory
Th17	IL-17, IL-21, IL-22, IL-25, IL-26	Pro-inflammatory
Th22	IL-22	Pro-inflammatory
Treg	TGFβ, IL-10, IL-35	Anti-inflammatory
Tfh	IFNγ, STAT3, IL-4, IL-10, IL-21	Help B cell activation
CTL	Perforin, Granzyme, INFγ, TNFα, TNFβ	Tumor cell/ Lyse infected
MC	TNFα, IL-1β, IL-6, IL-12, IL-23, MCP1, ROS	Pro-inflammatory

## Data Availability

No new data were created or analyzed in this study.

## Abbreviations

ACLF	Acute, chronic liver failure
ALD	Alcoholic liver disease
APCs	Antigen-presenting cells
bFGF	Basic fibroblast growth factor
CAID	Cirrhosis-associated immune dysfunction
CCL5	C-C motif chemokine ligand 5
CLIF-C	Chronic Liver Failure Consortium
COX	Cyclooxygenase
DAMPs	Danger-associated molecular patterns
EP2	Prostaglandin E receptor 2
FGF21	Fibroblast growth factor 21
FoxO	Forkhead box O
G-CSF	Granulocyte colony-stimulating factor
GM-CSF	Granulocyte-macrophage colony-stimulating factor
GPCRs	G-protein-coupled receptors
HBV-ACLF	Hepatitis B virus-related acute-on-chronic liver failure
HMG1	High mobility protein group 1
IFN-γ	Interferon-gamma
iNKT	Invariant killer T cells
KC	Kupffer cells
LTE4	Leukotriene E 4
LSEC	Liver sinusoidal endothelial cells
MELD	Model of End-Stage Liver Disease
MERTK	Mer receptor tyrosine kinase
NAFLD	Non-alcoholic fatty liver disease
NK	Natural killer
NLRs	NOD-like receptors
PAMPs	Pathogen-associated molecular patterns
PG	Prostaglandin
PGE2	Derived eicosanoid prostaglandin E2
PHLF	Post-hepatectomy liver failure
PRRs	Pattern recognition receptors
SCF	Stem cell factor
TLRs	Toll-like receptors
TIPS	Transjugular intrahepatic portal-systemic shunt
TGF-β1/IL-31	Transforming growth factor β1/interleukin-31
12-HHT	12-Hydroxyheptadecatrienoic acid

## References

[1] Kalaitzakis E. (2014). Gastrointestinal dysfunction in liver cirrhosis. World J. Gastroenterol..

[2] Noor M.T., Manoria P. (2017). Immune Dysfunction in Cirrhosis. J. Clin. Transl. Hepatol..

[3] Hernaez R., Kramer J.R., Liu Y., Tansel A., Natarajan Y., Hussain K.B., Ginès P., Solà E., Moreau R., Gerbes A. (2019). Prevalence and short-term mortality of acute-on-chronic liver failure: A national cohort study from the USA. J. Hepatol..

[4] Leão G.S., Lunardi F.L., Picon R.V., Tovo C.V., de Mattos A.A. (2019). Acute-on-chronic liver failure: A comparison of three different diagnostic criteria. Ann. Hepatol..

[5] Sarin S.K., Choudhury A. (2016). Acute-on-chronic liver failure: Terminology, mechanisms and management. Nat. Rev. Gastroenterol. Hepatol..

[6] Robinson M.W., Harmon C., O’farrelly C. (2016). Liver immunology and its role in inflammation and homeostasis. Cell. Mol. Immunol..

[7] Albillos A., Martin-Mateos R., Van der Merwe S., Wiest R., Jalan R., Álvarez-Mon M. (2022). Cirrhosis-associated immune dysfunction. Nat. Rev. Gastroenterol. Hepatol..

[8] Brandl K., Kumar V., Eckmann L. (2017). MINI-REVIEW Microbiome and Host Interactions Gut-liver axis at the frontier of host-microbial interactions. Am. J. Physiol. Gastrointest. Liver. Physiol..

[9] Bilzer M., Roggel F., Gerbes A.L. (2006). Role of Kupffer cells in host defense and liver disease. Liver Int..

[10] Sana G., Lombard C., Vosters O., Jazouli N., Andre F., Stephenne X., Smets F., Najimi M., Sokal E.M. (2014). Adult Human Hepatocytes Promote CD4^+^ T-Cell Hyporesponsiveness Via Interleukin-10-Producing Allogeneic Dendritic Cells. Cell Transplant..

[11] Wen Y., Lambrecht J., Ju C., Tacke F. (2021). Hepatic macrophages in liver homeostasis and diseases-diversity, plasticity and therapeutic opportunities. Cell Mol. Immunol..

[12] Furze R.C., Rankin S.M. (2008). Neutrophil mobilization and clearance in the bone marrow. Immunology.

[13] Gu X., Chu Q., Ma X., Wang J., Chen C., Guan J., Ren Y., Wu S., Zhu H. (2022). New insights into iNKT cells and their roles in liver diseases. Front. Immunol..

[14] Zhang I.W., López-Vicario C., Duran-Güell M., Clària J. (2021). Mitochondrial Dysfunction in Advanced Liver Disease: Emerging Concepts. Front. Mol. Biosci..

[15] Zaccherini G., Weiss E., Moreau R. (2021). Acute-on-chronic liver failure: Definitions, pathophysiology and principles of treatment. JHEP Rep..

[16] Moreau R., Gao B., Papp M., Bañares R., Kamath P.S. (2021). Acute-on-chronic liver failure: A distinct clinical syndrome. J. Hepatol..

[17] Bhatti G.K., Gupta A., Pahwa P., Khullar N., Singh S., Navik U., Kumar S., Mastana S.S., Reddy A.P., Reddy P.H. (2022). Targeting mitochondrial bioenergetics as a promising therapeutic strategy in metabolic and neurodegenerative diseases. Biomed. J..

[18] Peiseler M., Schwabe R., Hampe J., Kubes P., Heikenwälder M., Tacke F. (2022). Immune mechanisms linking metabolic injury to inflammation and fibrosis in fatty liver disease—Novel insights into cellular communication circuits. J. Hepatol..

[19] Zhang I.W., Curto A., López-Vicario C., Casulleras M., Duran-Güell M., Flores-Costa R., Colsch B., Aguilar F., Aransay A.M., Lozano J.J. (2022). Mitochondrial dysfunction governs immunometabolism in leukocytes of patients with acute-on-chronic liver failure. J. Hepatol..

[20] Yang X., Lu D., Zhuo J., Lin Z., Yang M., Xu X. (2020). The Gut-liver Axis in Immune Remodeling: New insight into Liver Diseases. Int. J. Biol. Sci..

[21] Guan H., Zhang X., Kuang M., Yu J. (2022). The gut–liver axis in immune remodeling of hepatic cirrhosis. Front. Immunol..

[22] Konturek P.C., Harsch I.A., Konturek K., Schink M., Konturek T., Neurath M.F., Zopf Y. (2018). Gut–Liver Axis: How Do Gut Bacteria Influence the Liver?. Med. Sci..

[23] Martin-Mateos R., Alvarez-Mon M., Albillos A. (2019). Dysfunctional Immune Response in Acute-on-Chronic Liver Failure: It Takes Two to Tango. Front. Immunol..

[24] Yunna C., Mengru H., Lei W., Weidong C. (2020). Macrophage M1/M2 polarization. Eur. J. Pharmacol..

[25] Clària J., Arroyo V., Moreau R. (2023). Roles of systemic inflammatory and metabolic responses in the pathophysiology of acute-on-chronic liver failure. JHEP Rep..

[26] Zhang L., Hu J., Gou C., Jin H., Zhang C., Liu Y., Wang Y., Wang X. (2022). Serum Interleukins as Potential Prognostic Biomarkers in HBV-Related Acute-on-Chronic Liver Failure. Mediat. Inflamm..

[27] Mantovani A., Dinarello C.A., Molgora M., Garlanda C. (2019). Interleukin-1 and Related Cytokines in the Regulation of Inflammation and Immunity. Immunity.

[28] Clària J., Arroyo V., Moreau R. (2016). The Acute-on-Chronic Liver Failure Syndrome, or When the Innate Immune System Goes Astray. J. Immunol..

[29] Hirano T. (2019). IL-6 in inflammation, autoimmunity and cancer. Int. Immunol..

[30] Karatayli E., Hall R.A., Weber S.N., Dooley S., Lammert F. (2019). Effect of alcohol on the interleukin 6-mediated inflammatory response in a new mouse model of acute-on-chronic liver injury. Biochim. Et Biophys. Acta Mol. Basis Dis..

[31] Clària J., Stauber R.E., Coenraad M.J., Moreau R., Jalan R., Pavesi M., Amorós, Titos E., Alcaraz-Quiles J., Oettl K. (2016). Systemic inflammation in decompensated cirrhosis: Characterization and role in acute-on-chronic liver failure. Hepatology.

[32] Dirchwolf M., Podhorzer A., Marino M., Shulman C., Cartier M., Zunino M., Paz S., Muñoz A., Bocassi A., Gimenez J. (2016). Immune dysfunction in cirrhosis: Distinct cytokines phenotypes according to cirrhosis severity. Cytokine.

[33] Zanza C., Romenskaya T., Manetti A.C., Franceschi F., La Russa R., Bertozzi G., Maiese A., Savioli G., Volonnino G., Longhitano Y. (2022). Cytokine Storm in COVID-19: Immunopathogenesis and Therapy. Medicina.

[34] Xiang X., Hwang S., Feng D., Shah V.H., Gao B. (2020). Interleukin-22 in alcoholic hepatitis and beyond. Hepatol. Int..

[35] Cron R.Q., Goyal G., Chatham W.W. (2023). Cytokine Storm Syndrome. Annu. Rev. Med..

[36] Remmler J., Schneider C., Treuner-Kaueroff T., Bartels M., Seehofer D., Scholz M., Berg T., Kaiser T. (2018). Increased Level of Interleukin 6 Associates with Increased 90-Day and 1-Year Mortality in Patients with End-Stage Liver Disease. Clin. Gastroenterol. Hepatol..

[37] Xiao N., Liu L., Zhang Y., Nie Y., Zhu X. (2022). A practical nomogram based on serum interleukin-6 for the prognosis of liver failure. Front. Med..

[38] Labenz C., Toenges G., Huber Y., Nagel M., Marquardt J.U., Schattenberg J.M., Galle P.R., Labenz J., Wörns M. (2019). Raised serum Interleukin-6 identifies patients with liver cirrhosis at high risk for overt hepatic encephalopathy. Aliment. Pharmacol. Ther..

[39] Tornai D., Mitchell M., McClain C.J., Dasarathy S., McCullough A., Radaeva S., Kroll-Desrosiers A., Lee J., Barton B., Szabo G. (2023). A novel score of IL-13 and age predicts 90-day mortality in severe alcohol-associated hepatitis: A multicenter plasma biomarker analysis. Hepatol. Commun..

[40] Wang S., Zhu H., Pan L., Zhang M., Wan X., Xu H., Hua R., Zhu M., Gao P. (2023). Systemic inflammatory regulators and risk of acute-on-chronic liver failure: A bidirectional mendelian-randomization study. Front. Cell Dev. Biol..

[41] Choy E.H., De Benedetti F., Takeuchi T., Hashizume M., John M.R., Kishimoto T. (2020). Translating IL-6 biology into effective treatments. Nat. Rev. Rheumatol..

[42] Xiang X., Feng D., Hwang S., Ren T., Wang X., Trojnar E., Matyas C., Mo R., Shang D., He Y. (2020). Interleukin-22 ameliorates acute-on-chronic liver failure by reprogramming impaired regeneration pathways in mice. J. Hepatol..

[43] Hwang S., Hicks A., Hoo C.Z., Kwon Y.S., Cho Y.E., Moore J., Gao B. (2023). Novel treatment of acute and acute-on-chronic liver failure: Interleukin-22. Liver Int..

[44] Arab J.P., Sehrawat T.S., Simonetto D.A., Verma V.K., Feng D., Tang T., Dreyer K., Yan X., Daley W.L., Sanyal A. (2020). An Open-Label, Dose-Escalation Study to Assess the Safety and Efficacy of IL-22 Agonist F-652 in Patients with Alcohol-associated Hepatitis. Hepatology.

[45] Cook S.A. (2023). The Pathobiology of Interleukin 11 in Mammalian Disease is Likely Explained by its Essential Evolutionary Role for Fin Regeneration. J. Cardiovasc. Transl. Res..

[46] Ng B., Xie C., Su L., Kuthubudeen F.F., Kwek X.-Y., Yeong D., Pua C.J., Cook S.A., Lim W.-W. (2023). IL11 (Interleukin-11) Causes Emphysematous Lung Disease in a Mouse Model of Marfan Syndrome. Arter. Thromb. Vasc. Biol..

[47] Widjaja A.A., Singh B.K., Adami E., Viswanathan S., Dong J., D’agostino G.A., Ng B., Lim W.W., Tan J., Paleja B.S. (2019). Inhibiting Interleukin 11 Signaling Reduces Hepatocyte Death and Liver Fibrosis, Inflammation, and Steatosis in Mouse Models of Nonalcoholic Steatohepatitis. Gastroenterology.

[48] Widjaja A.A., Viswanathan S., Shekeran S.G., Adami E., Lim W.-W., Chothani S., Tan J., Goh J.W.T., Chen H.M., Lim S.Y. (2022). Targeting endogenous kidney regeneration using anti-IL11 therapy in acute and chronic models of kidney disease. Nat. Commun..

[49] Gustavsson M. (2020). New insights into the structure and function of chemokine receptor: Chemokine complexes from an experimental perspective. J. Leukoc. Biol..

[50] Hughes C.E., Nibbs R.J.B. (2018). A guide to chemokines and their receptors. FEBS J..

[51] Lei W., Jia L., Wang Z., Liang Z., Zhao A., Liu Y., Tian Y., Zhao L., Chen Y., Shi G. (2023). CC chemokines family in fibrosis and aging: From mechanisms to therapy. Ageing Res. Rev..

[52] Khanam A., Trehanpati N., Riese P., Rastogi A., Guzman C.A., Sarin S.K. (2017). Blockade of Neutrophil’s Chemokine Receptors CXCR1/2 Abrogate Liver Damage in Acute-on-Chronic Liver Failure. Front. Immunol..

[53] Matsushima K., Yang D., Oppenheim J.J. (2022). Interleukin-8: An evolving chemokine. Cytokine.

[54] Wang Y.-H., Wang M.-L., Tao Y.-C., Wu D.-B., Chen E.-Q., Tang H. (2023). The high level of IL-1β in the serum of ACLF patients induces increased IL-8 expression in hUC-MSCs and reduces the efficacy of hUC-MSCs in liver failure. Stem Cell Res. Ther..

[55] Sasaki T., Suzuki Y., Kakisaka K., Wang T., Ishida K., Suzuki A., Abe H., Sugai T., Takikawa Y. (2019). IL-8 induces transdifferentiation of mature hepatocytes toward the cholangiocyte phenotype. FEBS Open Bio.

[56] Liu L., Chen P., Xiao N., Liu Q., Zhu X. (2023). Interleukin-8 predicts short-term mortality in acute-on-chronic liver failure patients with hepatitis B-related-related cirrhosis background. Ann. Med..

[57] Zhu B., Gao F., Li Y., Shi K., Hou Y., Chen J., Zhang Q., Wang X. (2023). Serum cytokine and chemokine profiles and disease prognosis in hepatitis B virus-related acute-on-chronic liver failure. Front. Immunol..

[58] Xiao L., Tang S., Zhang L., Ma S., Zhao Y., Zhang F., Xie Z., Li L. (2021). Serum CXCL1 Is a Prognostic Factor for Patients with Hepatitis B Virus–Related Acute-On-Chronic Liver Failure. Front. Med..

[59] Tang S., Zhang J., Zhang L., Zhao Y., Xiao L., Zhang F., Li Q., Yang Y., Liu Q., Xu J. (2023). Knockdown of CXCL1 improves ACLF by reducing neutrophil recruitment to attenuate ROS production and hepatocyte apoptosis. Hepatol. Commun..

[60] Liu G., Wang X., Yang T., Yan Y., Xiang T., Yang L., Luo X. (2022). High Interleukin-8 Levels Associated with Decreased Survival in Patients with Cirrhosis Following Transjugular Intrahepatic Portosystemic Shunt. Front. Med..

[61] Berres M.-L., Asmacher S., Lehmann J., Jansen C., Görtzen J., Klein S., Meyer C., Strunk H.M., Fimmers R., Tacke F. (2015). CXCL9 is a prognostic marker in patients with liver cirrhosis receiving transjugular intrahepatic portosystemic shunt. J. Hepatol..

[62] Lehmann J.M., Claus K., Jansen C., Pohlmann A., Schierwagen R., Meyer C., Thomas D., Manekeller S., Claria J., Strassburg C.P. (2018). Circulating CXCL10 in cirrhotic portal hypertension might reflect systemic inflammation and predict ACLF and mortality. Liver Int..

[63] Huang M., Jiao J., Cai H., Zhang Y., Xia Y., Lin J., Shang Z., Qian Y., Wang F., Wu H. (2022). C-C motif chemokine ligand 5 confines liver regeneration by down-regulating reparative macrophage-derived hepatocyte growth factor in a forkhead box O 3a–dependent manner. Hepatology.

[64] Lazarus H.M., Pitts K., Wang T., Lee E., Buchbinder E., Dougan M., Armstrong D.G., Paine R., Ragsdale C.E., Boyd T. (2023). Recombinant GM-CSF for diseases of GM-CSF insufficiency: Correcting dysfunctional mononuclear phagocyte disorders. Front. Immunol..

[65] Lu W.-Y., Bird T.G., Boulter L., Tsuchiya A., Cole A.M., Hay T., Guest R.V., Wojtacha D., Man T.Y., Mackinnon A. (2015). Hepatic progenitor cells of biliary origin with liver repopulation capacity. Nat. Cell Biol..

[66] Simonetto D.A., Shah V.H., Kamath P.S. (2017). Improving survival in ACLF: Growing evidence for use of G-CSF. Hepatol. Int..

[67] Engelmann C., Habtesion A., Hassan M., Kerbert A.J., Hammerich L., Novelli S., Fidaleo M., Philips A., Davies N., Ferreira-Gonzalez S. (2022). Combination of G-CSF and a TLR4 inhibitor reduce inflammation and promote regeneration in a mouse model of ACLF. J. Hepatol..

[68] Yu X., Guo R., Ming D., Deng Y., Su M., Lin C., Li J., Lin Z., Su Z. (2015). The Transforming Growth Factor β1/Interleukin-31 Pathway Is Upregulated in Patients with Hepatitis B Virus-Related Acute-on-Chronic Liver Failure and Is Associated with Disease Severity and Survival. Clin. Vaccine Immunol..

[69] Ruiz-Margáin A., Pohlmann A., Ryan P., Schierwagen R., Chi-Cervera L.A., Jansen C., Mendez-Guerrero O., Flores-García N.C., Lehmann J., Torre A. (2018). Fibroblast growth factor 21 is an early predictor of acute-on-chronic liver failure in critically ill patients with cirrhosis. Liver Transplant..

[70] Narumiya S., Yokomizo T., Aoki J. (2017). Lipid Mediators in Inflammation. Inflammation—From Molecular and Cellular Mechanisms to the Clinic.

[71] Artru F., McPhail M.J.W., Triantafyllou E., Trovato F.M. (2022). Lipids in Liver Failure Syndromes: A Focus on Eicosanoids, Specialized Pro-Resolving Lipid Mediators and Lysophospholipids. Front. Immunol..

[72] López-Vicario C., Checa A., Urdangarin A., Aguilar F., Alcaraz-Quiles J., Caraceni P., Amorós A., Pavesi M., Gómez-Cabrero D., Trebicka J. (2020). Targeted lipidomics reveals extensive changes in circulating lipid mediators in patients with acutely decompensated cirrhosis. J. Hepatol..

[73] Sasaki F., Yokomizo T. (2019). The leukotriene receptors as therapeutic targets of inflammatory diseases. Int. Immunol..

[74] Casulleras M., Flores-Costa R., Duran-Güell M., Zhang I.W., López-Vicario C., Curto A., Fernández J., Arroyo V., Clària J. (2022). Albumin Lipidomics Reveals Meaningful Compositional Changes in Advanced Cirrhosis and Its Potential to Promote Inflammation Resolution. Hepatol. Commun..

[75] Becares N., Härmälä S., China L., Colas R.A., Maini A.A., Bennet K., Skene S.S., Shabir Z., Dalli J., O’brien A. (2020). Immune Regulatory Mediators in Plasma from Patients With Acute Decompensation Are Associated with 3-Month Mortality. Clin. Gastroenterol. Hepatol..

[76] O’Brien A.J., Fullerton J.N., A Massey K., Auld G., Sewell G., James S., Newson J., Karra E., Winstanley A., Alazawi W. (2014). Immunosuppression in acutely decompensated cirrhosis is mediated by prostaglandin E2. Nat. Med..

[77] Clària J., Curto A., Moreau R., Colsch B., López-Vicario C., Lozano J.J., Aguilar F., Castelli F.A., Fenaille F., Junot C. (2021). Untargeted lipidomics uncovers lipid signatures that distinguish severe from moderate forms of acutely decompensated cirrhosis. J. Hepatol..

[78] Huang X.-P., Wang Y., Chen L., Sun W., Huang Y., Xu Y., Feng T.-T., Luo E.-P., Qin A.-L., Zhao W.-F. (2017). Elevated serum prostaglandin E2 predicts the risk of infection in hepatitis B virus-related acute-on-chronic liver failure patients. Asian Pac. J. Trop. Med..

[79] Wang Y., Chen C., Qi J., Wu F., Guan J., Chen Z., Zhu H. (2019). Altered PGE2-EP2 is associated with an excessive immune response in HBV-related acute-on-chronic liver failure. J. Transl. Med..

[80] China L., Maini A., Skene S.S., Shabir Z., Sylvestre Y., Colas R.A., Ly L., Salles N.B., Belloti V., Dalli J. (2018). Albumin Counteracts Immune-Suppressive Effects of Lipid Mediators in Patients with Advanced Liver Disease. Clin. Gastroenterol. Hepatol..

[81] Wang J., Liu Y., Ding H., Shi X., Ren H. (2021). Mesenchymal stem cell-secreted prostaglandin E2 ameliorates acute liver failure via attenuation of cell death and regulation of macrophage polarization. Stem Cell Res. Ther..

[82] Campbell A., Dronfield M., Toghill P., Reeves W. (1981). Neutrophil function in chronic liver disease. Clin. Exp. Immunol..

[83] Tritto G., Bechlis Z., Stadlbauer V., Davies N., Francés R., Shah N., Mookerjee R.P., Such J., Jalan R. (2011). Evidence of neutrophil functional defect despite inflammation in stable cirrhosis. J. Hepatol..

[84] DeMeo A.N., Andersen B.R., English D.K., Peterson J. (1971). Defective Chemotaxis Associated with a Serum Inhibitor in Cirrhotic Patients. J. Lab. Clin. Med..

[85] Van Epps D.E., Strickland R.G., Williams R.C. (1975). Inhibitors of leukocyte chemotaxis in alcoholic liver disease. Am. J. Med..

[86] Mookerjee R.P., Stadlbauer V., Lidder S., Wright G.A., Hodges S.J., Davies N.A., Jalan R. (2007). Neutrophil dysfunction in alcoholic hepatitis superimposed on cirrhosis is reversible and predicts the outcome. Hepatology.

[87] Fiuza C., Salcedo M., Clemente G., Tellado J.M. (2002). Granulocyte Colony-Stimulating Factor Improves Deficient In Vitro Neutrophil Transendothelial Migration in Patients with Advanced Liver Disease. Clin. Vaccine Immunol..

[88] Kedarisetty C.K., Anand L., Bhardwaj A., Bhadoria A.S., Kumar G., Vyas A.K., David P., Trehanpati N., Rastogi A., Bihari C. (2015). Combination of Granulocyte Colony-Stimulating Factor and Erythropoietin Improves Outcomes of Patients with Decompensated Cirrhosis. Gastroenterology.

[89] Rolando N., Wade J., Davalos M., Wendon J., Philpott-Howard J., Williams R. (2000). The Systemic Inflammatory Response Syndrome in Acute Liver Failure. Hepatology.

[90] Konstantis G., Tsaousi G., Pourzitaki C., Kitsikidou E., Magouliotis D.E., Wiener S., Zeller A.C., Willuweit K., Schmidt H.H., Rashidi-Alavijeh J. (2023). Efficacy of Granulocyte Colony-Stimulating Factor in Acute on Chronic Liver Failure: A Systematic Review and Survival Meta-Analysis. J. Clin. Med..

[91] Hou X., Li Y., Yuan H., Cai J., Liu R., Li J., Zhu C. (2021). Therapeutic Effect and Safety of Granulocyte Colony-Stimulating Factor Therapy for Acute-On-Chronic Liver Failure: A Systematic Review and Meta-Analysis of Randomized Controlled Trials. Front. Med..

[92] Choe W.H., Baik S.K. (2015). Prostaglandin E2-mediated immunosuppression and the role of albumin as its modulator. Hepatology.

[93] Arroyo V., Moreau R. (2014). Tying up PGE2 with albumin to relieve immunosuppression in cirrhosis. Nat. Med..

[94] Bernsmeier C., Pop O.T., Singanayagam A., Triantafyllou E., Patel V.C., Weston C.J., Curbishley S., Sadiq F., Vergis N., Khamri W. (2015). Patients With Acute-on-Chronic Liver Failure Have Increased Numbers of Regulatory Immune Cells Expressing the Receptor Tyrosine Kinase MERTK. Gastroenterology.

[95] Kou K., Sun X., Tian G., Zhi Y., Fan Z., Lv G. (2022). The Mechanisms of Systemic Inflammatory and Immunosuppressive Acute-on-Chronic Liver Failure and Application Prospect of Single-Cell Sequencing. J. Immunol. Res..

[96] Li Z.-H., Chen J.-F., Zhang J., Lei Z.-Y., Wu L.-L., Meng S.-B., Wang J.-L., Xiong J., Lin D.-N., Wang J.-Y. (2023). Mesenchymal Stem Cells Promote Polarization of M2 Macrophages in Mice with Acute-On-Chronic Liver Failure via Mertk/JAK1/STAT6 Signaling. Stem Cells.

[97] Lian Z.X., Li L. (2020). The Liver as a Lymphoid Organ. Liver Immunology: Principles and Practice.

[98] Weiss E., de la Grange P., Defaye M., Lozano J.J., Aguilar F., Hegde P., Jolly A., Moga L., Sukriti S., Agarwal B. (2021). Characterization of Blood Immune Cells in Patients With Decompensated Cirrhosis Including ACLF. Front. Immunol..

[99] Jeong W.-I., Park O., Suh Y.-G., Byun J.-S., Park S.-Y., Choi E., Kim J.-K., Ko H., Wang H., Miller A.M. (2011). Suppression of innate immunity (natural killer cell/interferon-γ) in the advanced stages of liver fibrosis in mice. Hepatology.

[100] Radaeva S., Sun R., Jaruga B., Nguyen V.T., Tian Z., Gao B. (2006). Natural Killer Cells Ameliorate Liver Fibrosis by Killing Activated Stellate Cells in NKG2D-Dependent and Tumor Necrosis Factor–Related Apoptosis-Inducing Ligand–Dependent Manners. Gastroenterology.

[101] Li H.-J., Yang N., Mu X., Tang L., Wang S.-S., Zhou C.-B., Yuan J.-H., Wang H.-Y., Yu Y.-Y., Li J. (2022). Reduction of natural killer cells is associated with poor outcomes in patients with hepatitis B virus-related acute-on-chronic liver failure. Hepatol. Int..

[102] Br V.K., Sarin S.K. (2023). Acute-on-chronic liver failure: Terminology, mechanisms and management. Clin. Mol. Hepatol..

[103] Wang N., He S., Zheng Y., Wang L. (2023). The value of NLR versus MLR in the short-term prognostic assessment of HBV-related acute-on-chronic liver failure. Int. Immunopharmacol..

[104] Sun L., Wang X., Saredy J., Yuan Z., Yang X., Wang H. (2020). Innate-adaptive immunity interplay and redox regulation in immune response. Redox Biol..

[105] Hensley M.K., Deng J.C. (2018). Acute on Chronic Liver Failure and Immune Dysfunction: A Mimic of Sepsis. Semin. Respir. Crit. Care Med..

[106] Mehta A.S., Long R.E., Comunale M.A., Wang M., Rodemich L., Krakover J., Philip R., Marrero J.A., Dwek R.A., Block T.M. (2008). Increased Levels of Galactose-Deficient Anti-Gal Immunoglobulin G in the Sera of Hepatitis C Virus-Infected Individuals with Fibrosis and Cirrhosis. J. Virol..

[107] Tangye S.G., Good K.L. (2007). Human IgM+CD27+ B Cells: Memory B Cells or “Memory” B Cells?. J. Immunol..

[108] Cook R.T., Waldschmidt T.J., Cook B.L., Labrecque D.R., Mclatchie K. (1996). Loss of the CD5+ and CD45RAhi B cell subsets in alcoholics. Clin. Exp. Immunol..

[109] Hassnine A.A., Saber M.A., Fouad Y.M., Sarhan H., Elsayed M.M., Zaki Z.M., Abdelraheem E.M., Abdelhalim S.M., Elsayed A.M. (2023). Clinical study on the efficacy of hepatitis B vaccination in hepatitis C virus related chronic liver diseases in Egypt. Virus Res..

[110] Ashhab A.A., Rodin H., Campos M., Abu-Sulb A., Hall J.A., Powell J., Debes J.D. (2020). Response to hepatitis B virus vaccination in individuals with chronic hepatitis C virus infection. PLoS ONE.

[111] Morishima C., Di Bisceglie A.M., Rothman A.L., Bonkovsky H.L., Lindsay K.L., Lee W.M., Koziel M.J., Fontana R.J., Kim H., Wright E.C. (2012). Antigen-specific T lymphocyte proliferation decreases over time in advanced chronic hepatitis C. J. Viral Hepat..

[112] Bernsmeier C., Triantafyllou E., Brenig R., Lebosse F.J., Singanayagam A., Patel V.C., Pop O.T., Khamri W., Nathwani R., Tidswell R. (2017). CD14^+^ CD15^−^ HLA-DR^−^ myeloid-derived suppressor cells impair antimicrobial responses in patients with acute-on-chronic liver failure. Gut.

[113] Shen C., Yan W.-Z., Zhao C.-Y., Che H.-H., Liu X.-Y., Liu Z.-Z., Wang Y.-D., Wang W., Li M., Gao J. (2015). Increased CD4+CD25+ regulatory T cells correlate with poor short-term outcomes in hepatitis B virus-related acute-on-chronic liver failure patients. J. Microbiol. Immunol. Infect..

[114] Wu Z., Shi H., Zhang L., Shi H., Miao X., Chen L., Chen Y., Ma Y. (2023). Comparative analysis of monocyte-derived dendritic cell phenotype and T cell stimulatory function in patients with acute-on-chronic liver failure with different clinical parameters. Front. Immunol..

[115] Adenote A., Dumic I., Madrid C., Barusya C., Nordstrom C.W., Prada L.R. (2021). NAFLD and Infection, a Nuanced Relationship. Can. J. Gastroenterol. Hepatol..

[116] Li B., Zhang C., Zhan Y.-T. (2018). Nonalcoholic Fatty Liver Disease Cirrhosis: A Review of Its Epidemiology, Risk Factors, Clinical Presentation, Diagnosis, Management, and Prognosis. Can. J. Gastroenterol. Hepatol..

[117] Widjaja A.A., Chothani S.P., Cook S.A. (2020). Different roles of interleukin 6 and interleukin 11 in the liver: Implications for therapy. Hum. Vaccines Immunother..

